# Oxygen Permeable Dextran Nanogels as a Hemoglobin-Based Oxygen Carrier

**DOI:** 10.34133/bmr.0355

**Published:** 2026-04-27

**Authors:** Jaeeun Oh, Hyemin Cho, Kidong Kim, Young-Joon Park, Yun Suk Huh, Sejin Son

**Affiliations:** ^1^ Industry-Academia Interactive R&E Center for Bioprocess Innovation, Inha University, Incheon 22212, Republic of Korea.; ^2^ College of Pharmacy, Ajou University, Gyeonggi, Suwon, Republic of Korea.; ^3^ Department of Biological Sciences, Inha University, Incheon 22212, Republic of Korea.

## Abstract

Hemoglobin-based oxygen carriers have been extensively explored as oxygen delivery platform. Among them, encapsulation-based hemoglobin-based oxygen carriers face a key limitation: Dense matrices surrounding hemoglobin hinder the precise control of oxygen binding and release, thereby reducing oxygen delivery efficiency to target tissues. In this study, we developed a hemoglobin-loaded dextran-based nanogel (Hb@Dex-NG) via Schiff base cross-linking between oxidized dextran and 4-arm polyethylene glycol (PEG)-NH_2_ to address these limitations. This nanogel system constructed via cross-linking of oxidized dextran and PEG features a permeable internal structure that enables efficient oxygen exchange. Ultraviolet–visible spectroscopy and oxygen dissociation analyses confirmed that the encapsulated hemoglobin retained oxygen-binding capacity. Notably, increasing the PEG content during synthesis led to denser internal structure, which, in turn, slowed oxygen release rates—demonstrating the modulability of this system as a controllable oxygen delivery carrier. Moreover, Hb@Dex-NG exhibited markedly reduced nitric oxide scavenging activity and suppressed autoxidation, suggesting enhanced vascular compatibility and oxidative stability. Overall, Hb@Dex-NG offers a structurally adjustable and biocompatible platform that overcomes major challenges associated with encapsulation strategy of oxygen delivery carrier, presenting a promising candidate for safe and effective oxygen delivery platforms.

## Introduction

Oxygen supply imbalance plays a crucial role in the pathological progression of various diseases, particularly in conditions such as hypoxemia, sickle cell disease, and chronic pulmonary diseases, where tissue oxygen deficiency leads to severe physiological dysfunction [1,2]. Insufficient tissue oxygenation can result in impaired cellular metabolism, leading to various pathological changes including tissue necrosis, inflammatory responses, and compromised immune function, potentially culminating in organ failure and death. To address these challenges, oxygen delivery systems designed to efficiently supply and regulate oxygen to living tissues have emerged as a significant area of research. These systems can serve as artificial blood substitutes, facilitate tissue regeneration, treat hypoxemia, and deliver oxygen therapy for various diseases, particularly benefiting patients with limited blood supply [3,4].

In response to these needs, hemoglobin (Hb)-based oxygen delivery carriers (HBOCs) have been extensively studied [5,6]. Hb, the major oxygen-carrying protein of red blood cells (RBCs), is a tetrameric metalloprotein composed of 2 alpha and 2 beta subunits (α_2_β_2_), each containing a heme prosthetic group that binds oxygen through a central ferrous iron (Fe^2+^) [3,7]. While various forms of HBOCs have been developed because of their high oxygen-carrying capacity and biocompatibility, free Hb outside the protective environment of RBCs can cause severe adverse effects [8,9]. Cell-free Hb tetramers can dissociate into dimers and monomers in blood vessels, causing nephrotoxicity and increasing oxidative damage to surrounding tissues [10]. Moreover, once removed from the reductive and enzymatically protected environment of RBCs, Hb becomes vulnerable to autoxidation, where the heme iron (Fe^2+^) is spontaneously oxidized to Fe^3+^ due to exposure to molecular oxygen and reactive species in plasma. This process generates methemoglobin (metHb)—which is incapable of binding oxygen—and produces reactive oxygen species, exacerbating oxidative stress [11]. In addition, free Hb can bind with nitric oxide (NO), a crucial vasodilator, leading to vasoconstriction and hypertension, which can result in serious physiological complications [12,13].

To overcome these challenges, various Hb-based oxygen delivery strategies have been investigated, with chemical modification being one of the most prominent approaches. One example is Hemospan, a polyethylene glycol (PEG)–Hb conjugates, designed to prolong blood circulation and reduce vasoconstrictive side effects [14]. However, such chemical modification methods may alter the secondary structure of Hb during the process, impairing its oxygen-binding and release capabilities [15]. In addition, incomplete chemical modification can result in the presence of free Hb, which may penetrate the vascular endothelium and raise concerns regarding biosafety, such as induction of oxidative damage, proinflammatory responses, and NO depletion, all of which can contribute to vascular dysfunction and tissue injury [16].

Recently attention has turned to encapsulation strategies to address these limitations [17–19]. In particular, nanogel-based systems composed of nanosized cross-linked polymer structures can offer modular structural properties and hold great promise as a next-generation platform for oxygen delivery. Nanogels are highly hydrated, 3-dimensional polymer structures with a permeable internal structure that allows for the encapsulation of biomolecules such as Hb without chemical modification. Their soft and flexible matrix provides a protective environment that preserves the structural integrity and function of Hb while preventing denaturation or oxidative degradation [20–22].

**Fig. 1.**
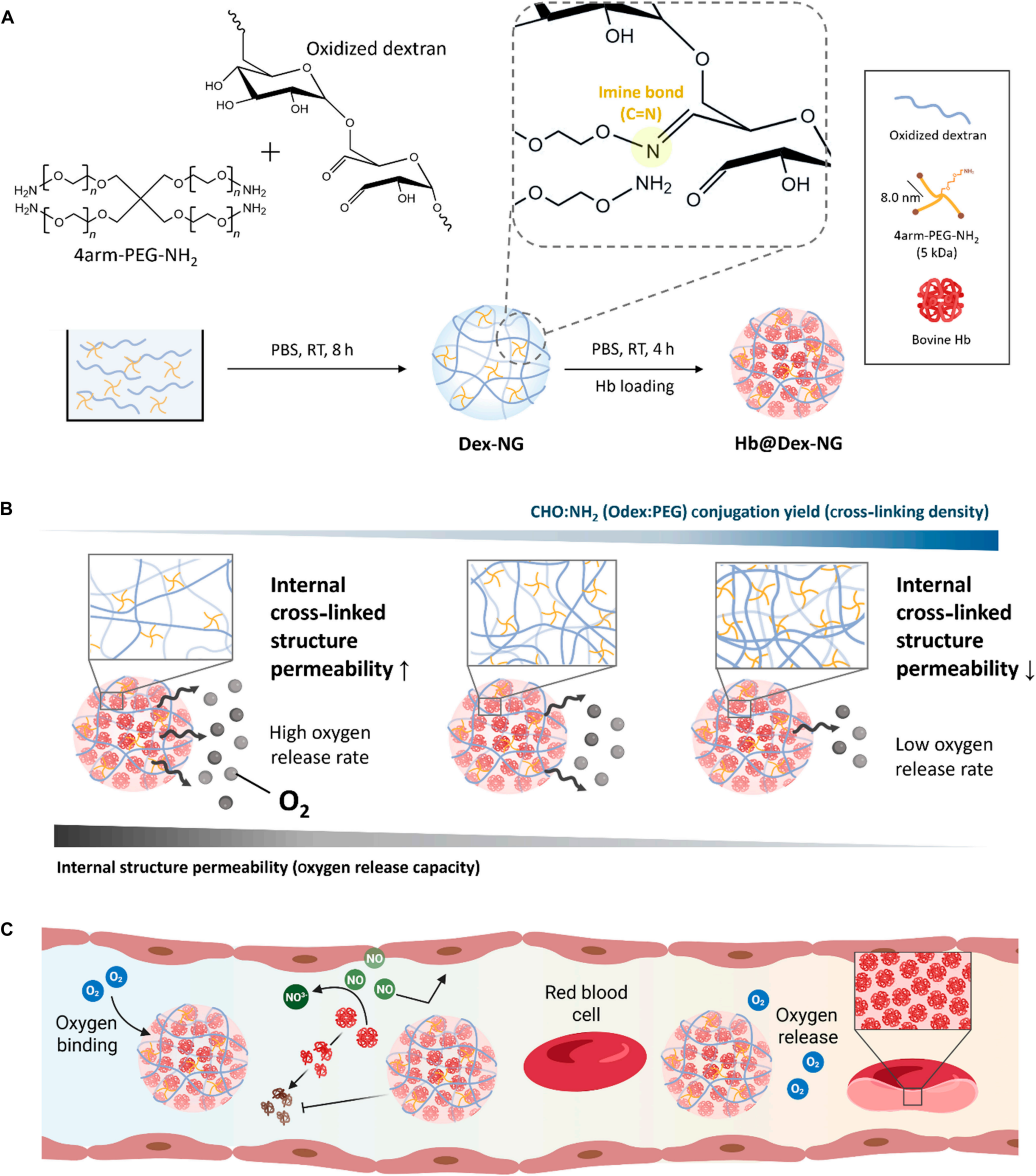
Schematic overview of hemoglobin-loaded dextran-based nanogel (Hb@Dex-NG). (A) Synthetic process of Hb@Dex-NG via Schiff base reaction between oxidized dextran and 4-armed polyethylene glycol amine (4arm-PEG-NH_2_), followed by post-loading of Hb. (B) Influence of CHO:NH_2_ conjugation yield on nanogel internal cross-linking density and its subsequent effect on oxygen release behavior. Ratios in parentheses indicate the feed molar ratios of oxidized dextran to 4arm-PEG-NH_2_ during nanogel synthesis. (C) Conceptual illustration of Hb@Dex-NG function in oxygen delivery.

Most of all, conventional encapsulation strategies often suffer from limited oxygen transfer efficiency—both in oxygen loading and release—due to the rigid outer barrier of the nanoparticle matrix. To address this key limitation, we developed Hb-loaded nanogel system (Hb@Dex-NG) that can control over internal cross-linked structure by modulating the degree of cross-linking of dextran backbone by 4-armed PEG amine (4arm-PEG-NH_2_) to enhance oxygen diffusion (Fig. 1A and B). Dextran, a biocompatible hydrophilic polysaccharide composed of d-glucose units, possesses abundant hydroxyl groups enabling various chemical modifications. Moreover, dextran exhibits remarkable hemocompatibility and is widely utilized as a plasma expander and anticoagulant due to its efficacy in preventing thrombosis and shock [23–25].

The 4arm-PEG-NH_2_, characterized by high biocompatibility, serves a dual role in nanogel synthesis: Its terminal amine groups react with aldehyde groups of oxidized dextran (Odex) to form covalent bonds, while its hydrophilic polymeric backbone contributes to the formation of a hydrated cross-linked structure. This dual functionality enables 4arm-PEG-NH_2_ to simultaneously serve as both a chemical cross-linking agent and a structural scaffold that augments the mechanical flexibility and hydration capacity of the resultant nanogel system [26]. Furthermore, the nanogels can encapsulate Hb without chemical modification, preserving its physiological function while protecting it from oxidative degradation, minimizing rapid clearance or leakage, and reducing toxicity by preventing direct contact with surrounding tissues and blood [27,28]. Collectively, these attributes contribute to the enhanced biocompatibility, functional stability, and safety of the Hb@Dex-NGs, underscoring its potential as a versatile oxygen delivery carrier (Fig. 1C).

## Materials and Methods

### Materials

Dextran (molecular weight [MW] = 40 kDa; catalog no. D1448) was obtained from Tokyo Chemical Industry (Japan). Sodium periodate (NaIO_4_; catalog no. 769517), Schiff’s fuchsin-sulfite reagent (catalog no. S5133), Hb from bovine blood (catalog no. H2500), diethylamine NONOate sodium salt hydrate (DEA-NONOate; catalog no. D184), *N*-(1-naphthyl)ethylenediamine dihydrochloride (catalog no. 33461), sulfanilamide (catalog no. S9251), fluorescamine (catalog no. F9015), sodium hydrosulfite (Na_2_S_2_O_4_; catalog no. 71699), tricarbonyldichlororuthenium(II) dimer (CORM-2; catalog no. 288144), and dimethyl sulfoxide (catalog no. 276855) were purchased from Sigma-Aldrich (St. Louis, MO, USA). Phosphate-buffered saline (PBS) (pH 7.2; catalog no. 20012-027) was purchased from Gibco, Thermo Fisher Scientific (Waltham, MA, USA). Amicon Ultra centrifugal filter units (MW cutoff = 50 kDa; catalog no. UFC805024) were obtained from Merck Millipore (Burlington, MA, USA). 4arm-PEG-NH_2_ (MW = 2 and 5 kDa; catalog no. A7032F and A7020) was obtained from JenKem Technology Co. Ltd. (Beijing, China). Potassium cyanide (KCN; catalog no. 6571-4405) was obtained from Daejung (Siheung, Republic of Korea). Potassium ferricyanide [K_3_Fe(CN)_6_; catalog no. 462] was purchased from Duksan (Ansan, Republic of Korea).

### Cells

The NIH/3T3 (CRL-1658) cells were obtained from Chonnam University (Gwangju, Korea) and maintained in Dulbecco’s modified Eagle’s medium, high-glucose medium (catalog no. 11965, Gibco) supplemented with 10% fetal bovine serum (FBS; catalog no. 12483-020, Gibco) and 1% penicillin–streptomycin (catalog no. 15140-122, Gibco). Female C57BL/6 mice (7 to 9 weeks old) were purchased from Orient Bio (Seongnam, Republic of Korea) and anesthetized using 2% isoflurane (Hana Pharm Co. Ltd.). Bone marrow cells were isolated and cultured in Dulbecco’s modified Eagle’s medium supplemented with 10% heat-inactivated FBS and 1% penicillin–streptomycin. Cells were differentiated into bone-marrow-derived macrophages (BMDMs) by treatment with macrophage colony-stimulating factor (20 ng/ml; catalog no. 51112-MNAH, Sino Biological). On day 2 or 3 of culture, half of the medium was gently removed and added with fresh medium. After 4 to 6 d of incubation, loosely adherent and adherent cells were collected by gentle detachment. The differentiated BMDMs were then counted and used for the following experiments. RBCs were isolated from female C57BL/6 mice. Female C57BL/6 mice (7 to 9 weeks old) was anesthetized using 2% isoflurane. Mouse whole blood obtained 800 μl per mouse use with a 26-gauge syringe via cardiac puncture, and the animals were euthanized by cervical dislocation immediately after blood withdrawal. Mouse whole blood was collected into EDTA-coated tubes and gently inverted. The blood samples were centrifuged at 1,500 rpm for 15 min at 4 °C to remove plasma and the buffy coat, as well as the RBC pellet. The RBC pellet was washed 3 times with cold PBS. Mouse-derived samples were obtained from the Institutional Animal Care and Use Committee of Inha University (approval number: INHA 230420-869-2). The samples were collected on 2025 April 16 and used until 2025 June 10.

### Synthesis of Hb@Dex-NG

#### Preparation of Odex

To oxidize dextran and generate aldehyde functional groups, dissolved in 2.5 ml of double-distilled water (ddH_2_O) with various concentrations (0.025, 0.08, or 0.2 M) of sodium periodate solution [29]. The mixture reacted for 1 h with gentle shaking at 4 °C under dark conditions. The oxidation product was purified using a PD-10 column equilibrated with ddH_2_O and collected in 2 ml fraction. The purified Odex was lyophilized to obtain a white powder (yield, 80%). The resulting Odex was stored at 4 °C in the dark until further use. ^1^H-nuclear magnetic resonance (NMR) spectra were recorded at room temperature (RT) using 850-MHz cryoprobe NMR spectrometer (Avance III HD 850, Bruker, Germany) with a sample concentration of 46 mM.

#### Aldehyde determination (Schiff’s test)

The oxidation degree was analyzed using Schiff’s test method. Briefly, the lyophilized Odex was dissolved in ddH_2_O to prepare a 22 mM (4 mg/ml) solution, and the solution was dispensed into each well of a 96-well plate. After adding 250 μl Schiff’s reagent to each well, the mixture was thoroughly mixed. The complex was analyzed spectrophotometrically at 535 nm within 20 min using ultraviolet–visible (UV–VIS) spectrophotometer (Synergy H1, BioTek Instruments Inc., USA). A glutaraldehyde-based calibration curve was used to measure aldehyde concentration.

#### Preparation of Dex-NGs

Dextran-based nanogels (Dex-NGs) were prepared as follows: Lyophilized Odex was dispersed in PBS (5 mg/ml), and then it was mixed with predetermined amount of 4arm-PEG-NH_2_ based on the desired feed molar ratio and stirred for 8 h at RT. The unreacted Odex and 4arm-PEG-NH_2_ were removed 6 times by a centrifugal filtration with centrifugal filter (MW cutoff = 50 kDa) at 14,000*g*: 3 min (first), 2 min (second), and 1.5 min (third to sixth) at RT. During early formulation development, an extended reaction step (up to 12 h total) was tested but was later adjusted during formulation optimization.

The conjugation between Odex and 4arm-PEG-NH_2_ was characterized using Fourier transform infrared (FT-IR) spectroscopy (FT/IR 6600, JASCO, Japan). Spectra were recorded in the range of 650 to 2,500 cm^−1^.

To quantitatively assess the conjugation yield between aldehyde (CHO) groups of Odex and amine (NH_2_) groups of 4arm-PEG-NH_2_, a fluorescamine assay was used. Following Dex-NG synthesis, the reaction mixture was subjected to ultracentrifugation (12,000 rpm for 30 min) to separate unreacted 4arm-PEG-NH_2_ in the supernatant. Supernatant was combined with prepared fluorescamine (3 mg/ml in dimethyl sulfoxide) and incubated at RT for 15 min under dark conditions. Fluorescence was measured using a UV–VIS spectrophotometer at excitation and emission wavelengths of 365 and 470 nm, respectively.

#### Preparation of Hb@Dex-NGs

After Dex-NG synthesis, 100 μl of purified Dex-NG solution was collected, to which 25 μl of bovine Hb solution (PBS, 250 mg/ml) was added and stirred for 4 h at RT. Hb@Dex-NGs were collected by ultracentrifugation (12,000 rpm for 10 min) and washed 3 times using PBS. For each wash, 500 μl of PBS was added to the pellet, followed by centrifugation at 12,000 rpm for 3 min.

### Characterization of Hb@Dex-NG

#### Size, zeta potential, and morphology

Nanogel size and zeta potential were measured using a dynamic light scattering (DLS) instrument (Nano ZS, Malvern, UK). After purification, 40 μl of nanogel suspension in PBS was used for size measurement. For zeta potential analysis, 810 μl of PBS was added to the same 40 μl of purified nanogel solution to make a total volume of 850 μl. Data reported represent *n* = 3 for each group. The morphology was observed under transmission electron microscopy (TEM; CM200, Philips, Netherlands).

### Hb loading efficiency measurement

The level of loaded Hb was assessed indirectly by measuring the concentration of Hb (Hb_total_) initially and then in the supernatant (Hb_free_) after ultracentrifugation. The loading efficiency of Hb was calculated as follows:

$$\text{Loading efficiency}\;\left(\%\right)=[\left({\mathrm{Hb}}_{\text{total}}-{\mathrm{Hb}}_{\text{free}}\right)/{\mathrm{Hb}}_{\text{total}}]\times 100\%$$ (1)

#### Colloidal stability measurement

Hb@Dex-NGs (15 mg/ml) were incubated in PBS and RPMI 1640 medium (Welgene) supplemented with varying concentrations of FBS (10, 25, and 50%) at 37 °C. Nanogel sizes were measured with DLS at predetermined time points (0, 0.17, 0.5, 1, 3, 6, 12, and 24 h) to evaluate short-term colloidal stability. For long-term colloidal stability, Hb@Dex-NGs were subjected to a 7 d incubation period during which nanogel size measurements were conducted at 24 h intervals.

### Oxygen-binding and -releasing state measurements

The oxygen-binding and -releasing capacity of Hb@Dex-NG (PBS, 15 mg/ml) monitored using a UV–VIS spectrophotometer. To generate deoxygenated Hb (deoxy-Hb), sodium dithionite was added to the Hb@Dex-NG at a 10:1 molar ratio relative to Hb monomer. For oxygenated Hb (oxy-Hb) formation, the deoxygenated solution was exposed to ambient oxygen for 5 min, resulting in complete conversion to the oxygenated form. The corresponding spectral shifts in the Soret absorption band to confirm the oxygen-binding state of Hb under each condition.

### Oxygen equilibrium curves

A Hemox analyzer (TCS Scientific Corp., USA) was used to measure oxygen-binding equilibria under simulated physiological conditions. RBCs and Hb@Dex-NG solution were prepared using the manufacturer-provided buffer and additive agents. The washing procedure for RBCs is described in the “Cells” section. Sodium dithionite was added to the Hb@Dex-NGs at a 20:1 molar ratio relative to the Hb monomer, followed by warming to 37 ± 0.05 °C and bubbling with air until a partial pressure of O_2_ (*p*O_2_) above 145 mmHg was reached. Absorbance data were collected, while the RBCs or Hb@Dex-NG solution was bubbled with nitrogen until it reached complete deoxygenation. Oxygen saturation curves were obtained by comparing the measured absorbance of the RBCs or Hb@Dex-NGs with the absorbance at a *p*O_2_ of 0 mmHg and the absorbance at maximum O_2_ saturation.

### Quantification of nitrite via Griess assay

NO levels were quantified indirectly by measuring nitrite (NO_2_^−^) concentration using the Greiss assay. Briefly, bovine Hb, Hemospan, and Hb@Dex-NGs were dissolved in PBS and tested at final Hb concentrations ranging from 10 to 320 μM. Each sample was combined with DEA-NONOate (100 μM) in 96-well plates. Afterward, sulfanilamide solution (10 mg/ml in 5% phosphoric acid) was added and incubated at RT for 5 min in the dark. Then, *N*-(1-naphthyl)ethylenediamine dihydrochloride solution (1 mg/ml in ddH_2_O) was added, and the mixture was incubated again under the same conditions. The absorbance was measured at 535 nm using a UV–VIS spectrophotometer.

### MetHb quantification

MetHb were quantified spectrophotometrically using chemical conversion methods. Bovine Hb, Hemospan, and Hb@Dex-NGs (PBS, 7 mg/ml) were incubated at 37 °C for predetermined time intervals (1, 1.5, 2, 2.5, 3, and 3.5 h). At each time point, aliquots were treated with 10% KCN and/or 10% K_3_Fe(CN)_6_, followed by incubation for 10 min at RT in the dark. The absorbance (*A*) at 630 nm was measured using a UV–VIS spectrophotometer. The MetHb content was calculated as follow:

$$\begin{array}{c} \begin{array}{c} \text{MetHb}\;\left(\%\right)=[\left(A_{\text{sample}}-A_{\mathrm{KCN}}\right)/\left(A_{K_{3}\mathrm{Fe}{\left(\mathrm{CN}\right)}_{6}}-A_{\mathrm{KCN}+K_{3}\mathrm{Fe}{\left(\mathrm{CN}\right)}_{6}}\right)]\times 100\% \end{array} \end{array}$$ (2)

### Hemolysis assay

The washing procedure for RBCs is described in the “Cells” section. RBC suspension (0.5%, v/v) was treated with PBS, ddH_2_O, and each concentration of Hb@Dex-NGs for 12 h at 37 °C. The RBCs were then centrifuged to collect supernatants. The absorbance (*A*) of released Hb from RBCs was measured at 540 nm using a UV–VIS spectrophotometer. Hemolysis rate (%) was calculated as follows:

$$\begin{array}{c} \text{Hemolysis rate}\;\left(\%\right)=[\left(A_{\text{sample}}-A_{\mathrm{PBS}}\right)/\left(A_{{\mathrm{ddH}}_{2}O}-A_{\mathrm{PBS}}\right)]\times 100\% \end{array}$$ (3)

### CCK-8 and ELISA assays

Cytotoxicity was assessed using the BMDM and NIH/3T3 cell line. Briefly, the cells were seeded in 96-well plate of 1 × 10^5^ cells (BMDMs) or 4 × 10^3^ cells (NIH/3T3 cells) per well and incubated with various concentrations (each containing Hb at 0.5, 1, 2.5, 5, 10, 20 mg/ml) of Hb@Dex-NGs for 24 h. After treatment, 200 μl of Cell Counting Kit-8 (CCK-8) solution in CCK-8 (Dojindo Laboratories) was added to each well and incubated for 1 h (BMDMs) or 20 min (NIH/3T3 cells) at 37 °C. Absorbance was measured at 450 nm using a UV–VIS spectrophotometer. BMDM supernatant collected from each well was analyzed to quantify the levels of tumor necrosis factor-α (TNF-α) by enzyme-linked immunosorbent assays (ELISAs) (Thermo Fisher Scientific, USA).

### Statistical analysis

All data are presented as means ± standard deviation (SD). GraphPad Prism version 8.0.2 (GraphPad Software, San Diego, CA, USA) was used for all statistical analyses.

## Results and Discussion

### Synthesis and characterization of Dex-NGs

**Fig. 2.**
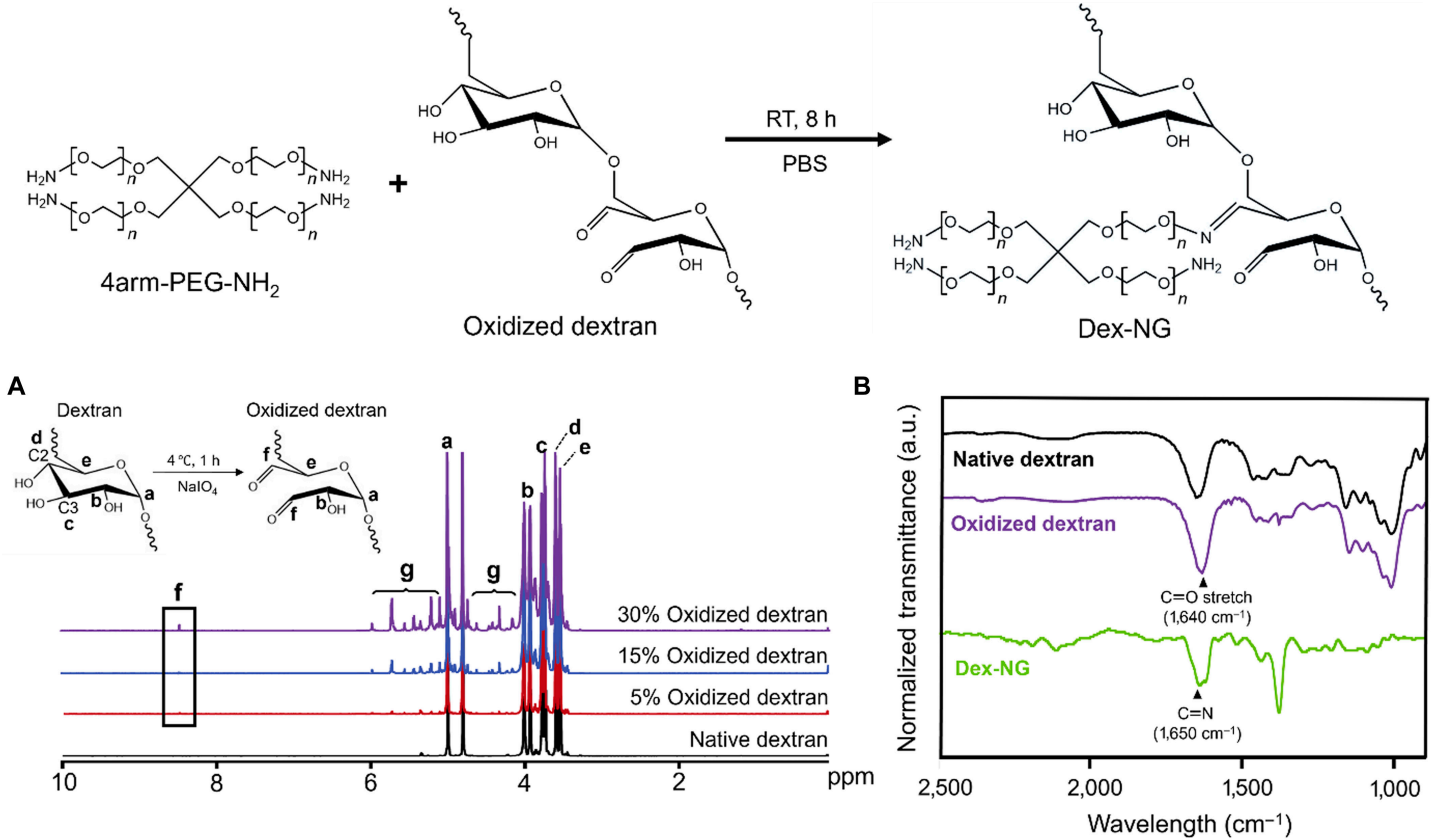
Structural characterization of dextran-based nanogel (Dex-NG) and its components. (A) ^1^H-nuclear magnetic resonance (NMR) spectra of native dextran and oxidized dextran with varying oxidation degrees. (B) Fourier transform infrared (FT-IR) spectra showing characteristic absorption bands: carboxylate (C〓O) stretches at 1,640 cm^−1^ in native and oxidized dextran and an imine (C〓N) stretches at 1,650 cm^−1^ in Dex-NG, indicating successful Schiff base conjugation.

Dex-NGs were synthesized through Schiff base cross-linking chemistry involving aldehyde-functionalized Odex and 4arm-PEG-NH_2_. The cross-linking relies on the formation of reversible imine bonds through nucleophilic addition of primary amine groups to aldehyde moieties, resulting in the assembly of a 3-dimensional internal cross-linked nanogels with nanoscale dimensions. The resulting cross-linked architecture is schematically represented in Fig. 1A. Odex was synthesized by reacting dextran with sodium periodate (NaIO_4_), followed by purification of sodium periodate using PD-10 column and lyophilization. To verify the extent of dextran oxidation, we conducted ^1^H-NMR spectroscopy on native dextran and Odex with varying oxidation degrees (Fig. 2A). Native dextran displayed characteristic resonances at 3.5 to 4.1 parts per million (ppm) (peaks a to e), corresponding to the glucose backbone protons within the glucopyranose ring structure. Upon periodate-mediated oxidation, new resonances emerged in the 5.0 to 5.6 ppm region (peak g), assigned to hemiacetal protons formed at the C2 and C3 positions following ring opening of the glucopyranose units. In addition, a diagnostic aldehydic proton signal (peak f) appeared at 8.0 to 9.0 ppm, providing direct evidence of aldehyde group formation. The assignment of each peak corresponds to the labeled protons (peaks a to g) shown in the annotated glucose substructure in Fig. 2A. The integrated intensities of the oxidation-induced peaks (peaks f and g) increased in proportion to the theoretical oxidation degree (5%, 15%, and 30%), confirming the successful and controllable aldehyde functionalization of dextran.

The formation of chemically cross-linked PEG and dextran structure, synthesized by reacting Odex and 4arm-PEG-NH_2_ in PBS for 8 h, was confirmed through FT-IR spectroscopic analysis (Fig. 2B). Odex did not exhibit the characteristic aldehyde peak typically observed at ~1,730 cm^−1^, attributed to hemiacetal formation [30,31]. Both native and Odex showed a broad absorption band at ~1,640 cm^−1^, corresponding to overlapping contributions from carboxylate (C=O) stretches. Upon reaction with 4arm-PEG-NH_2_, this peak underwent a notable shift, accompanied by the emergence of a new absorption band at 1,650 cm^−1^, diagnostic of imine (C=N) bond formation. This confirmed the successful formation of Schiff base linkages of 4arm-PEG-NH_2_ and CHO–dextran for nanogel formation.

To systematically investigate the modularity of Dex-NG physicochemical properties, 3 key synthesis parameters were varied: (a) the MW of PEG (2 and 5 kDa), (b) the feed molar ratio CHO to NH_2_ (1:1, 2, and 4), and (c) the oxidation degree of dextran (5%, 15%, and 30%). The oxidation degree of dextran was defined as the percentage of anhydrous glucose units that were converted into aldehyde functionalities through periodate oxidation. The CHO:NH_2_ molar ratio represents the stoichiometric ratio of aldehyde groups of Odex to amine groups of 4arm-PEG-NH_2_, where higher NH_2_ reflects higher PEG content. These parameters were selected on the basis of their potential to modulate cross-linking density of internal cross-linked nanogels. As shown in Table 1, at a fixed oxidation degree (15%) of dextran, the PEG MW significantly influenced nanogel formation characteristics, including size, polydispersity index (PDI), and conjugation yield—defined as the percentage of aldehyde groups that participated in nanogel formation. At a CHO:NH_2_ ratio of 1:1, 2 kDa PEG (sample ID 4) showed a conjugation yield of 19.2%, forming nanogels with a size of 599 nm and a PDI of 0.18. In contrast, 5 kDa PEG (sample ID 7) exhibited a similar conjugation yield (20.0%) but produced smaller and more uniform nanogels (429 nm, PDI = 0.07). At a 1:2 ratio, 2 kDa PEG (sample ID 5) achieved a higher conjugation yield (37.9%) than 5 kDa PEG (sample ID 8, 23.1%) and formed significantly larger and more polydisperse nanogels (942 nm, PDI = 0.27 versus 542 nm, PDI = 0.14). At a 1:4 ratio, 2 kDa PEG (sample ID 6) resulted in a conjugation yield of 44.1%, with nanogels of 492 nm and a PDI of 0.42. Meanwhile, 5 kDa PEG (sample ID 9) showed a higher conjugation yield (71.2%) but formed nanogels of slightly larger size (568 nm) and higher PDI (0.52). This result suggests that although 2 kDa PEG efficiently reacts with aldehyde groups, its short chain length may be insufficient to form an extended cross-linked structure, leading to less uniform and loosely structured nanogels. In contrast, 5 kDa PEG can connect more distant aldehyde groups, allowing the formation of a more compact and uniform nanogel structure, even with a lower conjugation yield.

**Table 1.** Characterization of Dex-NGs

Sample ID	Dextran oxidation degree (%)	PEG MW (chain length/arm)	Feed molar ratio (CHO:NH_2_)	Size (nm)	PDI	Conjugation yield (%)	Zeta potential **(−mV)**
1	5	5 kDa (8.0 nm)	1:1	696.7	0.25 ± 0.05	9.0 %	−2.7 ± 1.4
2			1:2	433.0	0.41 ± 0.04	7.1 %	−5.6 ± 1.1
3			1:4	488.7	0.41 ± 0.04	6.3 %	−3.5 ± 0.8
4	15	2 kDa (3.2 nm)	1:1	599.1	0.18 ± 0.04	19.2 %	−7.5 ± 0.5
5			1:2	942.2	0.27 ± 0.05	37.9 %	−6.4 ± 0.4
6			1:4	492.4	0.42 ± 0.12	44.1 %	−5.5 ± 1.4
7		5 kDa (8.0 nm)	1:1	429.3	0.07 ± 0.04	20.0 %	−23.5 ± 0.7
8			1:2	541.7	0.14 ± 0.06	23.1 %	−10.7 ± 2.4
9			1:4	568.3	0.52 ± 0.23	71.2 %	−5.3 ± 1.7
10	30	5 kDa (8.0 nm)	1:1	443.0	0.12 ± 0.06	13.6 %	−4.1 ± 1.8
11			1:2	603.5	0.42 ± 0.08	18.8 %	−7.5 ± 3.1
12			1:4	380.9	0.67 ± 0.09	22.6 %	−13.5 ± 0.2

Dex-NGs, dextran-based nanogels; PEG, polyethylene glycol; MW, molecular weight; PDI, polydispersity index.

At a fixed oxidation degree (15%) and PEG MW (5 kDa), increasing the CHO:NH_2_ feed molar ratio from 1:1 to 1:4 (sample IDs 7 to 9) resulted in a marked increase in conjugation yield from 20.0% to 71.2%, indicating enhanced crosslink formation with higher amine availability. However, this was accompanied by an increase in nanogel size (429 to 568 nm) and PDI (0.07 to 0.52), indicating that excess PEG may hinder uniform nanogel structure formation despite improved cross-linking efficiency.

To exclude the effect of oxidation degree of dextran, we synthesized nanogels with 5 kDa PEG and a fixed CHO:NH_2_ ratio of 1:1. Increasing the oxidation degree from 5% to 15% led to increase in conjugation yield (from 9.0% to 20.0%) and a corresponding decrease in nanogel size (from 697 to 429 nm), along with improved uniformity (PDI from 0.25 to 0.07). However, further increasing the oxidation degree to 30% did not reduce the size further (443 nm) and resulted in higher PDI (0.12) and a lower conjugation yield (13.6%), indicating that excessive oxidation does not necessarily improve cross-linking efficiency. These findings demonstrate that the physicochemical characteristics of nanogel can be finely modulated by adjusting the PEG chain length, the CHO:NH_2_ feed ratio, and the oxidation degree of dextran, in particular, as to internal cross-linking density. Based on comprehensive physicochemical characterization, a representative Dex-NG formulation was selected for Hb encapsulation. The formulation prepared with 15% Odex, 4arm-PEG-NH_2_ (5 kDa), and a 1:2 CHO:NH_2_ ratio (sample ID 7) was identified as optimal due to its balanced properties: moderate nanogel size (361 nm), narrow size distribution (PDI = 0.08), and reproducible synthetic performance, collectively indicating stable and homogeneous nanogel formation.

**Fig. 3.**
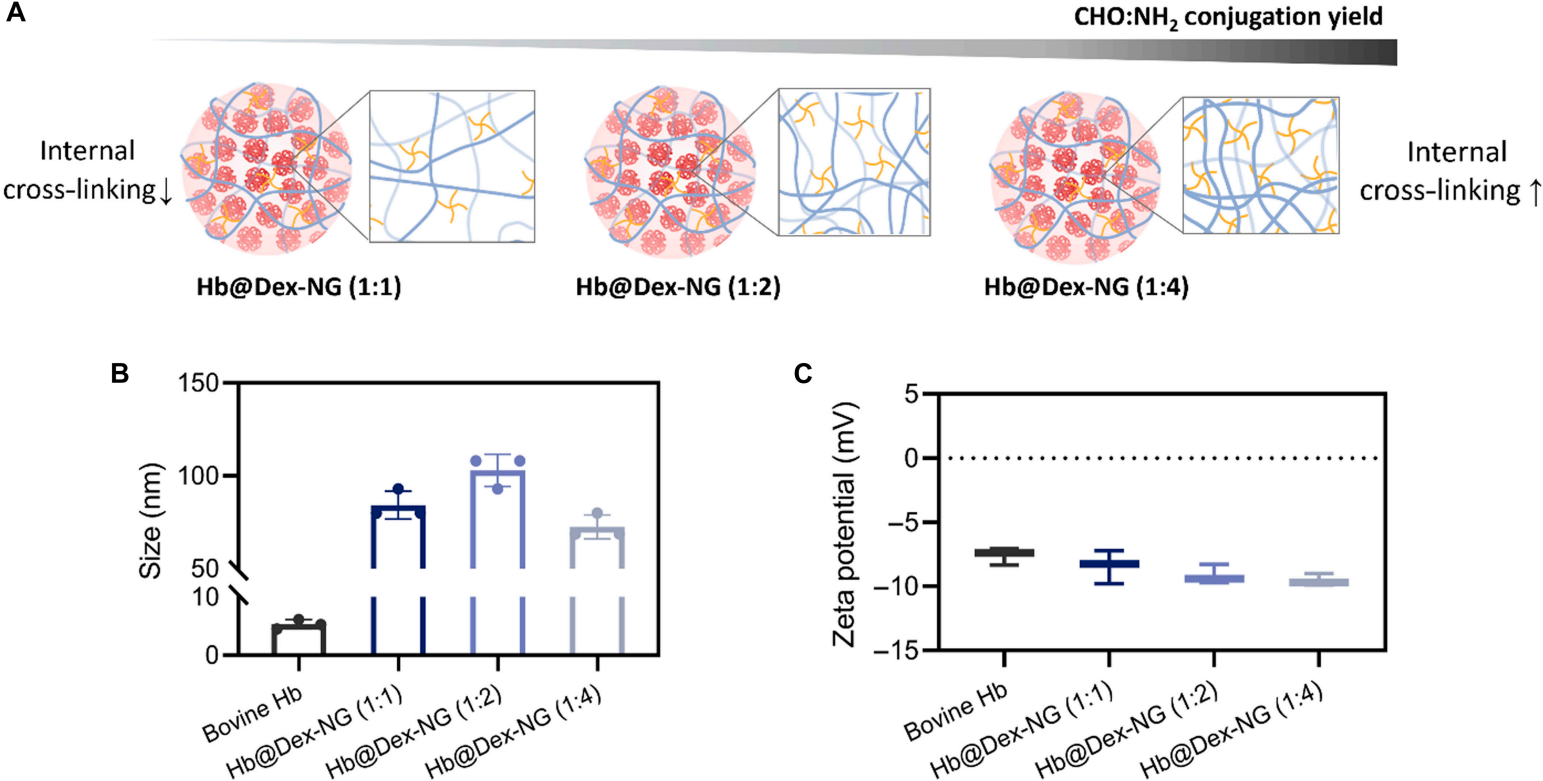
Physicochemical characterization of hemoglobin-loaded dextran-based nanogel (Hb@Dex-NG). (A) Schematic illustrations depict increasing internal cross-linking density and conjugation yield with higher polyethylene glycol (PEG) content. (B) Hydrodynamic size, (C) zeta-potential of cell-free Hb (bovine Hb), and Hb@Dex-NG formulations synthesized using CHO:NH_2_ feed molar ratios of 1:1, 1:2, and 1:4 by dynamic light scattering (DLS).

Following Hb encapsulation, the Hb@Dex-NG synthesized at a feed molar ratio of 1:1 (CHO:NH_2_) exhibited favorable physicochemical properties, with a nanogel size of 90 nm and a low PDI (0.18) (Fig. 3B). The hydrodynamic diameters of the 1:2 and 1:4 (CHO:NH_2_) formulations were 102 nm (PDI = 0.15) and 72 nm (PDI = 0.27), respectively. Zeta potential measurements showed values of −8.4, −9.3, and −9.5 mV for the 1:1, 1:2, and 1:4 groups, respectively (Fig. 3C). Conjugation yield analysis, quantified as the percentage of aldehyde groups participating in crosslink formation, revealed a positive correlation with PEG content: 21.6% (1:1), 35.4% (1:2), and 69.8% (1:4) (Table 2). This trend indicates that a higher concentration of PEG increases the number of available amine groups, which, in turn, improves the likelihood of forming covalent bonds with aldehyde groups on Odex, resulting in nanogels with denser internal cross-linking.

**Table 2.** Characterization of Hb@Dex-NGs

Group	Feed molar ratio (CHO:NH_2_)	Size (nm)	PDI	Zeta potential (−mV)	Conjugation yield (%)	Hb loading efficiency (%)
Cell-free Hb	–	5.31 nm	0.96 ± 0.04	−7.59 ± 0.5	–	–
Dex-NG (1:1)	1:1	361 nm	0.08 ± 0.01	−1.58 ± 0.7	21.6%	24.9%
Hb@Dex-NG (1:1)		90 nm	0.18 ± 0.02	−8.38 ± 1.4		
Dex-NG (1:2)	1:2	329 nm	0.37 ± 0.03	−4.37 ± 1.4	35.4%	21.4%
Hb@Dex-NG (1:2)		102 nm	0.15 ± 0.04	−9.31 ± 0.8		
Dex-NG (1:4)	1:4	361 nm	0.23 ± 0.03	−4.49 ± 1.5	69.8 %	18.3%
Hb@Dex-NG (1:4)		72 nm	0.27 ± 0.02	−9.51 ± 0.5		

Hb@Dex-NG, hemoglobin-loaded dextran-based nanogel; PDI, polydispersity index.

**Fig. 4.**
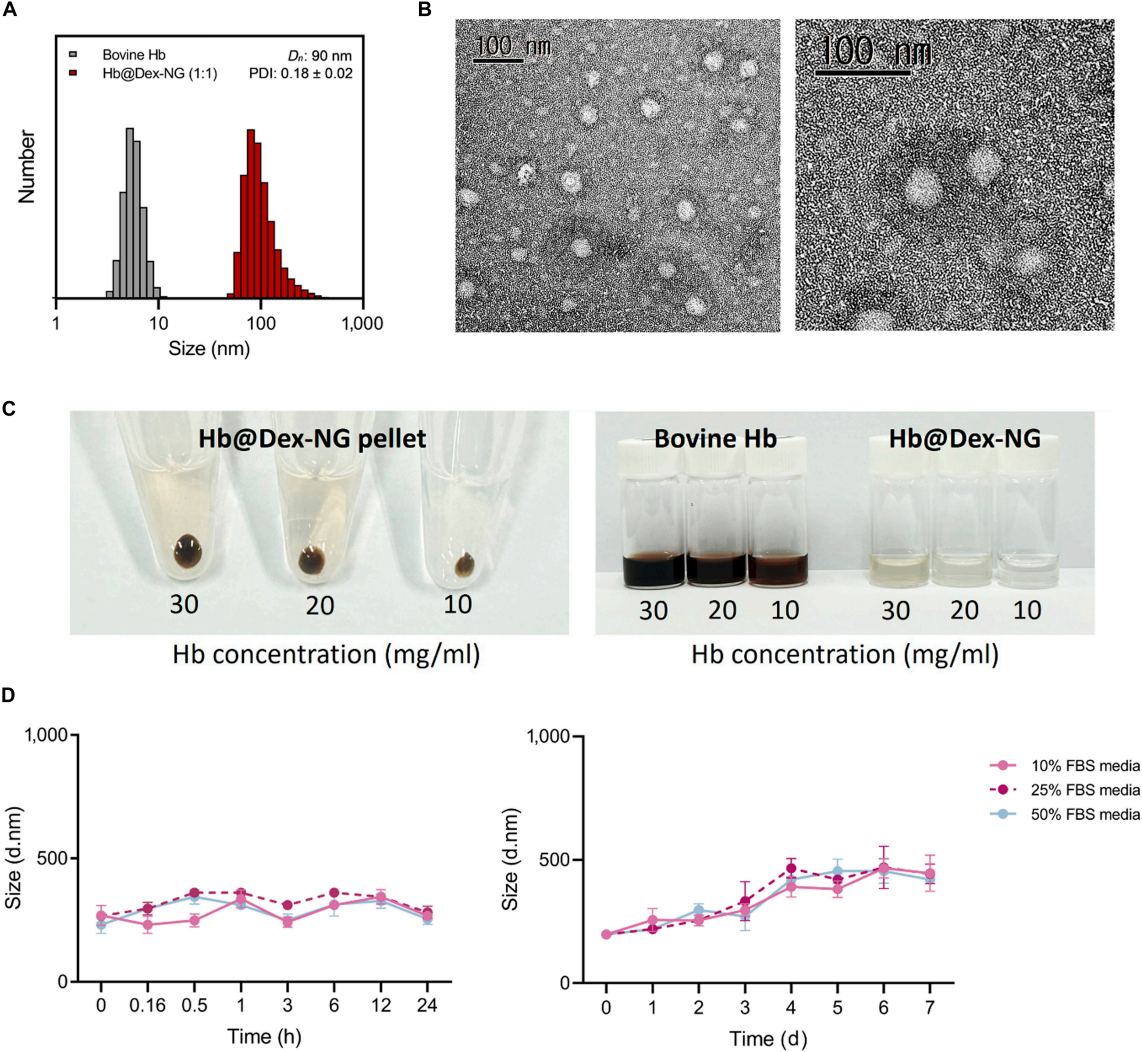
Detailed characterization of hemoglobin-loaded dextran-based nanogel (Hb@Dex-NG). (A) Dynamic light scattering (DLS) size distribution profiles of free Hb and Hb@Dex-NG (1:1). (B) Transmission electron microscopy (TEM) images of Hb@Dex-NG (1:1). (C) Visual assessment of Hb@Dex-NG pellet formation after ultracentrifugation and dispersion compared to free Hb solution at different Hb concentrations. (D) Short-term (0 to 24 h) and long-term (0 to 7 d) colloidal stability of Hb@Dex-NG in FBS-containing media (10%, 25%, and 50%), evaluated via nanogel size changes over time.

The 1:1 (CHO:NH_2_) feed molar ratio was selected as the optimal formulation for all subsequent experiments (Fig. 4A). While its physicochemical properties were comparable to those of the 1:2 formulation, the 1:1 ratio yielded the highest Hb loading efficiency among all tested formulations, a critical factor governing oxygen-carrying capacity. The TEM analysis of the Hb@Dex-NG revealed uniformly spherical without noticeable aggregation suggesting high morphological uniformity (Fig. 4B). The observed average diameter of 50 to 60 nm was smaller than the hydrodynamic diameter measured by DLS, which reflects the combined size of the nanogel and its associated hydration shell in aqueous dispersion. To assess the Hb encapsulation efficiency, we subjected Hb@Dex-NG dispersions prepared with increasing Hb concentrations (10, 20, and 30 mg/ml) to centrifugation. Distinct pellets were observed in the bottom of tubes at all tested concentrations (Fig. 4C, left), indicating successful entrapment of Hb within the nanogel matrix. While bovine Hb solutions of the same concentrations retained dark brown color, Hb@Dex-NG dispersions appeared light brown color (Fig. 4C, right), which attributed to the optical shielding effect of the dextran matrix surrounding the encapsulated Hb.

### Colloidal stability evaluation of Hb@Dex-NG

To assess the colloidal stability of Hb@Dex-NG under physiologically relevant conditions and compare its stability across varying serum concentrations, nanogel size changes using DLS under physiologically relevant conditions (Fig. 4D). Colloidal stability is defined as the ability of nanogels to maintain their size and dispersion without aggregation or precipitation over time. Intensity-based size distribution measurements were used because of spectral interference from serum proteins, which precluded accurate number-based size distribution analysis.

The colloidal stability of Hb@Dex-NG was assessed in RPMI 1640 medium containing various concentrations of FBS (10%, 25%, and 50%). Under all serum conditions, the nanogels exhibited relatively uniform nanogel sizes throughout the initial 24 h period, demonstrating robust short-term colloidal stability (Fig. 4D, left). Following extended incubation over 7 d, a modest increase in nanogel size was observed (Fig. 4D, right); however, this phenomenon was consistent across all FBS concentrations, suggesting that serum content exerted negligible influence on the long-term stability profile of the nanogels. These results confirm that Hb@Dex-NG maintains stable colloidal behavior in physiologically relevant media, irrespective of serum concentration.

### Oxygen delivery capacity of Hb@Dex-NG

The oxygen delivery capacity of Hb is a key property when developing HBOCs. To assess whether the encapsulated Hb within the nanogels retained its ability to bind and release oxygen, we used a UV–VIS spectrophotometer. Under oxygenated and deoxygenated conditions, the UV-vis absorption spectrum of Hb demonstrates characteristic spectral changes, most prominently in the Soret band (400 to 430 nm), which originates from π–π* transitions within the porphyrin ring and exhibits sensitivity to the coordination state of the heme iron [32]. Specifically, oxy-Hb exhibits a strong Soret peak at around 414 nm, whereas in the deoxy-Hb, this peak shifts to 430 nm. The complementary Q-band absorptions (500 to 600 nm) further provide distinct spectroscopic signatures that differentiate between oxygenated and deoxygenated states, with oxy-Hb exhibiting 2 characteristic Q-band peaks at 542 and 576 nm.

**Fig. 5.**
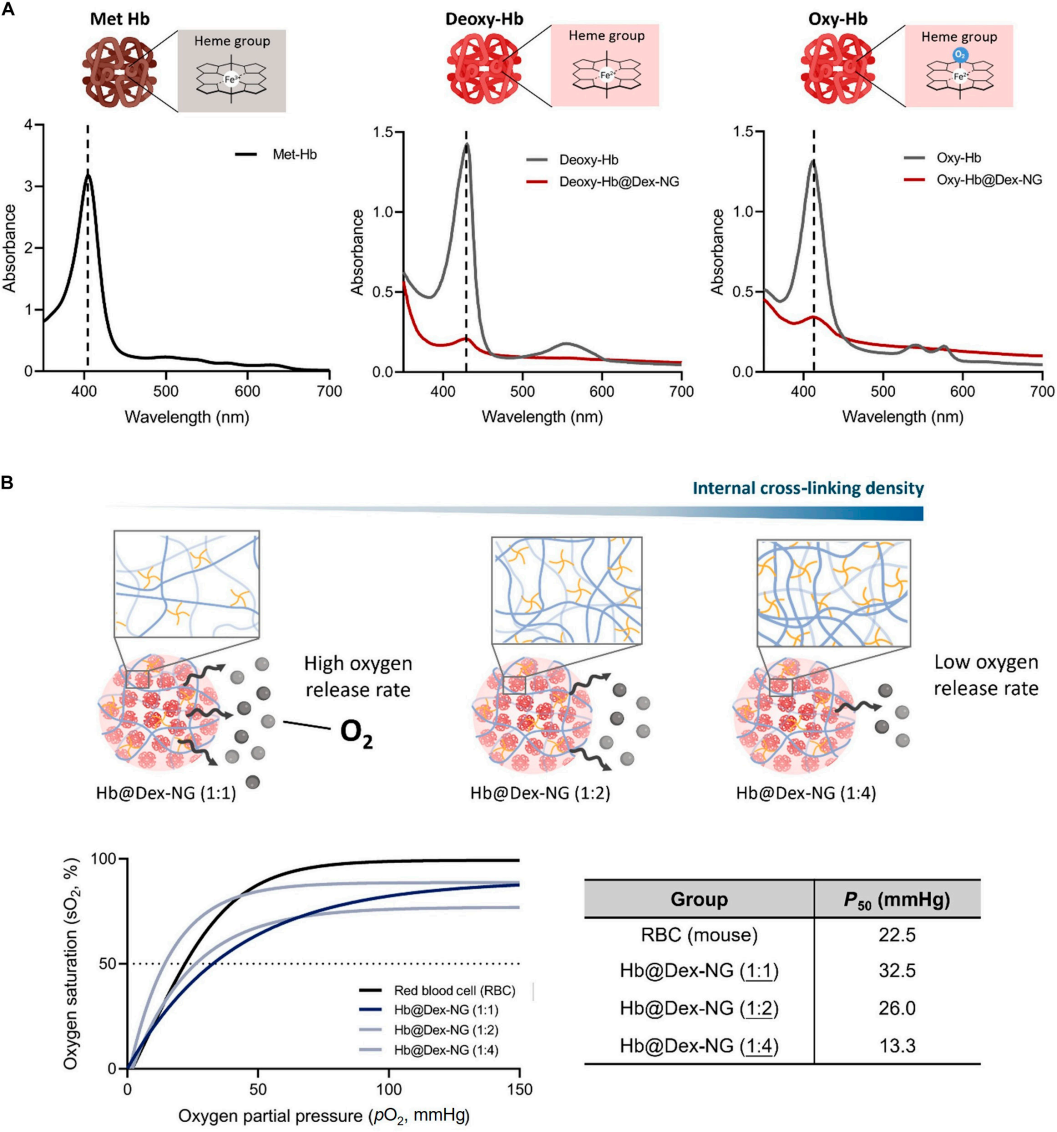
Evaluation of oxygen delivery capacity of hemoglobin-loaded dextran-based nanogel (Hb@Dex-NG). (A) Ultraviolet–visible (UV–vis) spectra of Hb@Dex-NG in oxygen-binding states, including deoxygenated Hb (deoxy-Hb) and oxygenated Hb (oxy-Hb). (B) Oxygen dissociation curves of Hb@Dex-NGs and red blood cells (RBCs) measured using a Hemox analyzer.

Figure 5A reveals that the Soret band of Hb@Dex-NGs underwent reversible wavelength shifts corresponding to under oxygenated and deoxygenated conditions, indicating that the encapsulated Hb retains its capability to reversibly bind and release oxygen. To induce deoxygenation, we treated samples with sodium dithionite, a chemical reducing agent that removes bound oxygen from Hb. Sodium dithionite treatment produced a characteristic deoxy-Hb peak at 430 nm, while reoxygenation through exposure to ambient air induced a spectral shift to 415 nm, corresponding to oxy-Hb. Consistent with previous studies on Hb-encapsulated nanoparticles, the UV-vis spectra of Hb@Dex-NGs exhibited a characteristic background slope attributed to light scattering by the nanoparticle matrix [33]. Although this scattering effect resulted in the attenuation or loss of certain Q-band absorption features, the distinctive Soret band remained readily identifiable, enabling the evaluation of Hb oxygen-binding and -releasing capacity within the nanogels. These spectral transitions confirm that encapsulated Hb retained its oxygen-binding and -releasing capacity comparable to free Hb, with no detectable impairment of heme spectroscopic properties.

Oxygen delivery capacity were quantitatively assessed through equilibrium curve analysis, correlating *p*O_2_ with the fractional oxygen saturation (*Y*) of Hb. Measurements were conducted using a Hemox analyzer at 37 °C, where Hb@Dex-NGs (10 mg/ml) were prepared in Hemox buffer, fully oxygenated by air bubbling (*p*O_2_ >145 mmHg) and then deoxygenated using nitrogen. Absorbance changes were recorded to construct oxygen saturation curves. The *P*_50_ parameter, representing the *p*O_2_ at which 50% oxygen saturation is achieved, provides a standardized measure of oxygen delivery capacity, where decreased *P*_50_ values correspond to increased binding strength. The observed hyperbolic profile is characteristic of bovine Hb, which exhibits lower intrinsic oxygen-binding cooperativity (Hill coefficient of 1.6 to 2.3) than native human Hb (Hill coefficient of 2.5 to 2.7) [34,35]. This indicates that the curve’s deviation from a classic sigmoidal shape is a baseline property of the protein itself.

As shown in Fig. 5B, mouse RBCs exhibited a *P*_50_ of 22.5 mmHg. The Hb@Dex-NGs showed *P*_50_ values of 32.5 mmHg (1:1), 26.0 mmHg (1:2), and 13.3 mmHg (1:4), respectively. A higher *P*_50_ (e.g., 32.5 mmHg at 1:1) indicates more rapid oxygen release, which may facilitate oxygen unloading to tissues. Notably, the 1:2 formulation exhibited a *P*_50_ (26.0 mmHg) closest to that of native RBCs, suggesting that this formulation may offers an oxygen release profile similar to that of native RBCs. In contrast, the markedly lower *P*_50_ observed at 1:4 (13.3 mmHg) implies slower oxygen release, potentially contributing to prolonged oxygen retention in circulation. Moreover, the *P*_50_ values decreased with increasing CHO:NH_2_ feed ratios. This phenomenon is likely due to the denser internal cross-linking of nanogels formed at elevated PEG content, as reflected by the increased conjugation yield (21.6% to 69.8%), which may hinder oxygen releasing and diffusion.

Importantly, compared to clinically investigated HBOCs, such as Hemospan (*P*_50_ ≈ 5 to 6 mmHg) [36] or Hemopure (*P*_50_ ≈ 40 ± 6 mmHg) [37], Hb@Dex-NGs exhibit structural modularity that enables precise control over oxygen release kinetics rather than intrinsic oxygen delivery capacity. This controllable nanogel architecture supports the rational design of oxygen carriers with modular oxygen release profiles tailored to different pathological demands.

### Prevention of NO scavenging through Hb encapsulation

Cell-free Hb readily reacts with NO, depleting this essential vasodilator and disrupting vascular homeostasis. This reaction leads to vasoconstriction and associated cardiovascular risks, thereby limiting the clinical applicability of HBOCs [38]. To evaluate NO scavenging activity, we indirectly quantified NO consumption through measurement of nitrite (NO_2_^−^) levels using the Griess assay. Hb@Dex-NGs, free Hb, and Hemospan were incubated with DEA-NONOate (100 μM) across various Hb concentrations (10–320 μM), and nitrite formation was assessed colorimetrically at 535 nm.

**Fig. 6.**
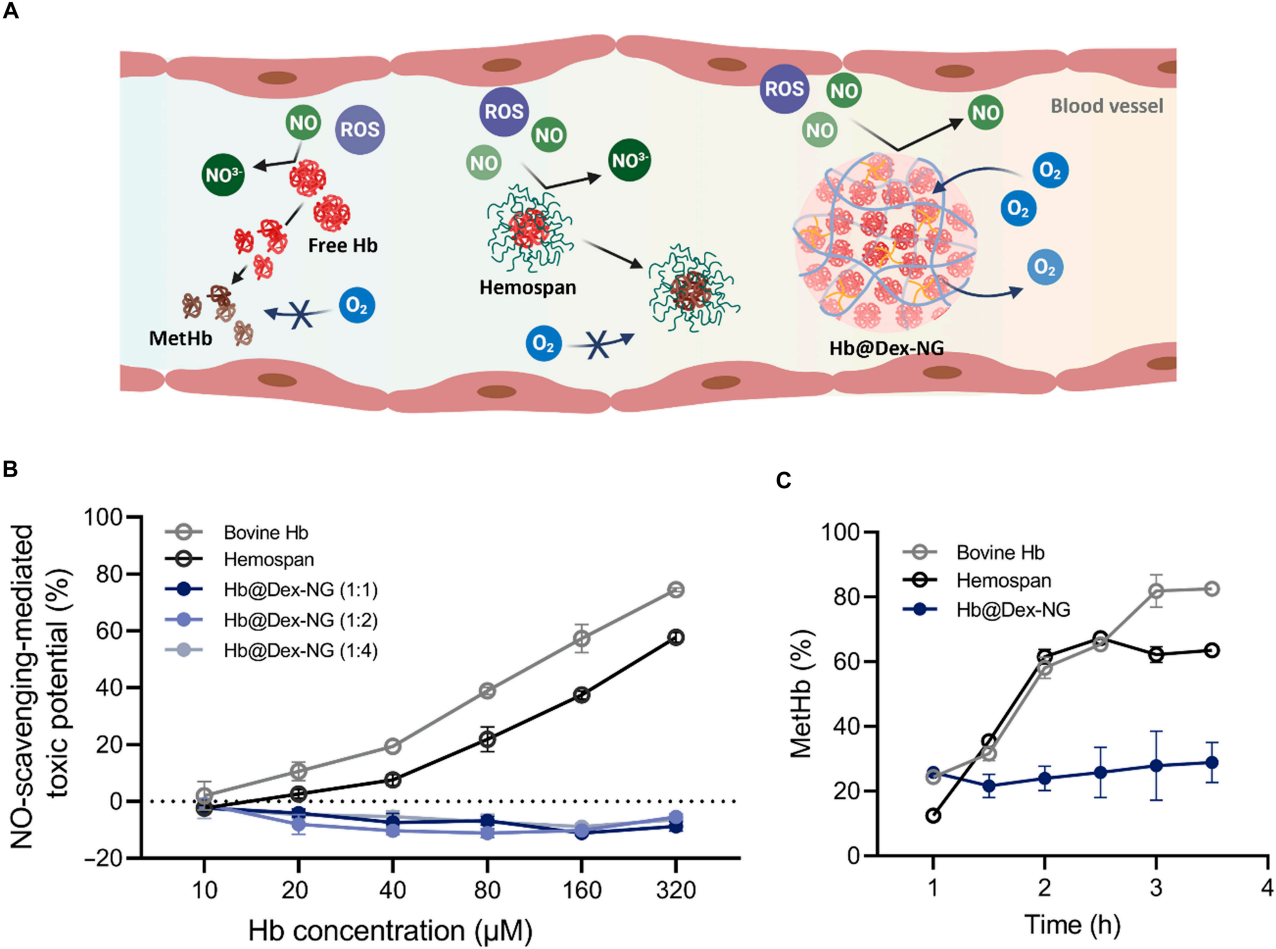
Nitric oxide (NO) scavenging activity and oxidative stability assessment of hemoglobin-loaded dextran-based nanogel (Hb@Dex-NG). (A) Schematic illustration of NO scavenging and reactive oxygen species (ROS)-induced oxidation by free Hb and Hemospan versus Hb@Dex-NG. (B) Quantitative analysis of NO scavenging activity by free Hb, Hemospan, and Hb@Dex-NG. (C) Quantitative analysis of time-dependent methemoglobin (metHb) formation in free Hb, Hemospan, and Hb@Dex-NG.

As shown in Fig. 6A and B, the free Hb and Hemospan demonstrated pronounced time-dependent NO consumption. Hemospan [14], a PEG-conjugated Hb formulation previously developed to enhance circulatory stability, still presents surface-exposed Hb molecules, rendering them susceptible to NO interaction. Conversely, all Hb@Dex-NG formulations across different CHO:NH_2_ molar ratios (1:1, 1:2, and 1:4) exhibited markedly reduced NO scavenging compared to free Hb and Hemospan. This protective effect demonstrates the effectiveness of the encapsulation approach, wherein Hb is physically sequestered within internally cross-linked dextran nanogels. The nanogel matrix functions as a selective molecular barrier that impedes NO diffusion kinetics, thereby reducing the probability of direct contact between NO molecules and heme iron centers. This diffusion interference mechanism effectively attenuates NO scavenging activity while preserving the reversible oxygen-binding and release capability. Notably, NO scavenging inhibition remained consistent across all nanogel formulations regardless of cross-linking density, indicating that the presence of an intact encapsulating barrier, rather than cross-linking extent, governs NO exclusion efficiency. These results demonstrate that Hb@Dex-NGs successfully address a fundamental challenge in Hb-based oxygen delivery by maintaining oxygen-binding capability while substantially reducing NO scavenging mediated toxicity.

### Inhibition of Hb autoxidation

Under physiological conditions, cell-free Hb readily undergoes autoxidation, converting Fe^2+^ heme to redox-inactive Fe^3+^ metHb, which is devoid of oxygen-binding capacity. This oxidative destabilization compromises oxygen carrying capacity and contributes to cytotoxicity effects, representing a major limitation for HBOCs [11]. Oxidative stability was assessed by monitoring metHb formation kinetics over time. The autoxidation rates were determined through measuring metHb formation. Each Hb formulation was incubated in PBS at 37 °C, and metHb levels were quantified spectrophotometrically at predetermined intervals.

As shown in Fig. 6A and C, free Hb underwent rapid oxidative degradation, accumulating over 40% metHb within 2 h under physiological conditions (PBS, pH 7.4, 37°C). Hemospan, despite PEG surface modification, demonstrated only marginal oxidative protection with metHb accumulation kinetics such as free Hb. This limited protective effect reflects the structural limitations of surface PEGylation, where polymer conjugation provides incomplete shielding of heme centers, leaving iron sites accessible to environmental oxidants.

In contrast, Hb@Dex-NGs demonstrated notably attenuated metHb formation, indicating enhanced oxidative stability through complete encapsulation within the cross-linked dextran matrix. The internal PEG cross-linking structure of the nanogel establishes an effective molecular barrier that diminishes oxidative attack on the heme iron, thereby preserving the ferrous oxidation state essential for oxygen binding. These findings demonstrate that nanogel encapsulation provides superior oxidative protection against autoxidation of Hb, establishing a more robust strategy for maintaining its oxygen delivery capacity under physiological conditions.

### Hemocompatibility evaluation

To ensure the safety of Hb@Dex-NGs intended for intravenous circulation, we evaluated the hemolytic potential of Hb@Dex-NGs by incubating RBCs at varying concentrations ranging from 0 to 50 mg/ml. These correspond to Hb equivalent concentrations of 0 to 40 mg/ml, selected to approximate physiologically relevant Hb concentrations in human blood (~150 mg/ml) while accommodating experimental constraints.

**Fig. 7.**
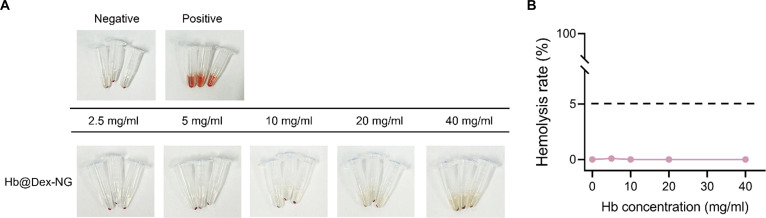
Hemolytic response assessment of hemoglobin-loaded dextran-based nanogel (Hb@Dex-NG). (A) Visual observation of red blood cells (RBCs) after incubation with various concentrations (0 to 50 mg/ml) of Hb@Dex-NG. Deionized water and phosphate-buffered saline (PBS) were used as positive and negative controls, respectively. (B) Hemolysis rate (%) of Hb@Dex-NG.

Figure 7 shows that after 12 h of incubation with the Hb@Dex-NGs, the hemolysis rates remained below 1% at all concentrations tested. To account for the optical contribution of residual nanogels in the supernatant, we quantified hemolysis by subtracting the absorbance of concentration-matched blank controls (Hb@Dex-NG without RBCs) from the experimental groups. According to internationally accepted criteria, materials exhibiting hemolysis rates below 5% are considered nonhemolytic, indicating that Hb@Dex-NGs demonstrate good hemocompatibility. These findings establish the superior blood compatibility of Hb@Dex-NGs and support their potential for clinical development as HBOCs. The dextran-based nanogel encapsulation strategy provides a biocompatible delivery platform that effectively mitigates hemolytic toxicity risks associated with extracellular or surface-accessible Hb, while maintaining therapeutic Hb concentrations suitable for oxygen delivery carriers.

### Cytotoxicity

To evaluate the cytotoxicity of the Hb@Dex-NG, we assessed cell viability using CCK-8 assays with varying concentrations of Hb@Dex-NGs ranging from 0.64 to 25.6 mg/ml in BMDMs and NIH/3T3 cells. Free Hb and Hemospan were included as comparative controls and applied at matched Hb-equivalent concentrations (Fig. S1A).

**Fig. 8.**
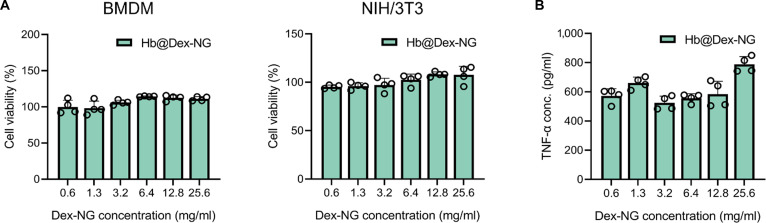
In vitro cytotoxicity assessment of hemoglobin-loaded dextran-based nanogel (Hb@Dex-NG). (A) Cell viability evaluation of Hb@Dex-NG in bone-marrow-derived macrophages (BMDMs) and NIH/3T3 cells after 24 h of incubation, assessed using a Cell Counting Kit-8 (CCK-8) assay. (B) Measurement of proinflammatory tumor necrosis factor-α (TNF-α) secretion from BMDMs treated with Hb@Dex-NG by enzyme-linked immunosorbent assay (ELISA).

As shown in Fig. 8A, Hb@Dex-NG maintained high cell viability in both cell types at all tested concentrations, indicating minimal cytotoxicity of the nanogel carrier. In BMDMs, all groups showed acceptable viability, with a slight increase in metabolic activity observed in the presence of Hb, possibly due to the stimulatory effects of iron-containing proteins on macrophage metabolism. In contrast, in NIH/3T3 cells, free Hb and Hemospan treatment led to a concentration-dependent decrease in viability, while Hb@Dex-NG preserved cell viability even at the highest concentration tested. These results confirm that the Hb@Dex-NG carrier itself does not induce cytotoxic effects under the tested conditions.

To assess additional immune-related cytotoxicity of the Hb@Dex-NG carrier, we measured TNF-α secretion by ELISA in BMDMs following treatment with Hb@Dex-NG (0.64 to 25.6 mg/ml) (Fig. 8B). TNF-α was used as a marker due to its relevance to Toll-like receptor 4-mediated inflammatory responses triggered by free Hb [39]. Across all tested concentrations, including the highest concentration (25.6 mg/ml), Hb@Dex-NG did not induce a marked increase in TNF-α secretion, indicating that the carrier does not trigger excessive inflammatory responses. Importantly, TNF-α levels induced by Hb@Dex-NG were comparable to those of Hemospan, which has undergone phase II clinical trials (Fig. S1B). This suggests that the immune response elicited by Hb@Dex-NG remains within a clinically acceptable range.

Collectively, the low cytotoxicity elicited by Hb@Dex-NG demonstrate a favorable biocompatibility profile. These findings underscore the translational potential of Hb@Dex-NG as an HBOC and support the need for comprehensive in vivo evaluation of its immunological safety

## Conclusion

In the present investigation, we have successfully engineered a novel Hb-encapsulated dextran-based nanogel that represents a significant advancement in oxygen delivery carriers, addressing critical limitations inherent to conventional HBOCs. The nanogel architecture was constructed via controlled Schiff base cross-linking between Odex and 4arm-PEG-NH_2_, forming an internally cross-linked nanogel in which the cross-linking density could be precisely modulated by adjusting the feed molar ratio of the 2 components.

Through comprehensive physicochemical characterization, the optimized Hb@Dex-NGs demonstrated controllable internal cross-linking density, which directly modulated the oxygen release kinetics—higher cross-linking density resulted in delayed oxygen release. The slower oxygen release observed in nanogels with higher cross-linking density was attributed to the diffusion-limiting effect of the nanogel internal structure, rather than any alteration in the intrinsic oxygen-binding properties of Hb. This controllable internal cross-linking density facilitated precise modulation of oxygen release behavior, as evidenced by systematic variations in *P*_50_ values. UV-vis spectrophotometry and oxygen dissociation kinetic analyses further confirmed that encapsulated Hb maintained its native oxygen delivery capacity.

Moreover, the Hb encapsulation strategy effectively addressed 2 major pathological mechanisms associated with free Hb—NO scavenging and autoxidation—through spatial confinement of Hb molecules within the nanogel matrix. In addition, separate in vitro evaluations revealed minimal hemolytic activity, no substantial cytotoxicity, and clinically acceptable levels of immune activation. These collective findings establish Dex-NGs as a promising platform for oxygen delivery carriers, offering simultaneous structural stabilization and functional optimization of oxygen transport capabilities.

The demonstrated efficacy of this dextran-based nanogel carrier approach warrants further investigation through comprehensive in vivo studies to evaluate oxygen delivery kinetics, pharmacokinetic profiles, biodistribution patterns, and long-term biosafety parameters. Such investigations will be essential to establish the clinical translatability and therapeutic efficacy of this next-generation HBOCs, potentially addressing unmet clinical needs in trauma medicine, surgical applications, and conditions characterized by compromised oxygen transport capacity.

## Data Availability

Data are available from the corresponding author upon request.
