# Supplementary material for: Oxygen Permeable Dextran Nanogels as a Hemoglobin-Based Oxygen Carrier
Source: Biomater Res. 2026 Apr 27;30:0355. doi: 10.34133/bmr.0355 (PMC13113307; doi:10.34133/bmr.0355)
Supplement: Supplementary 1 — Fig. S1 [file bmr.0355.f1.docx]

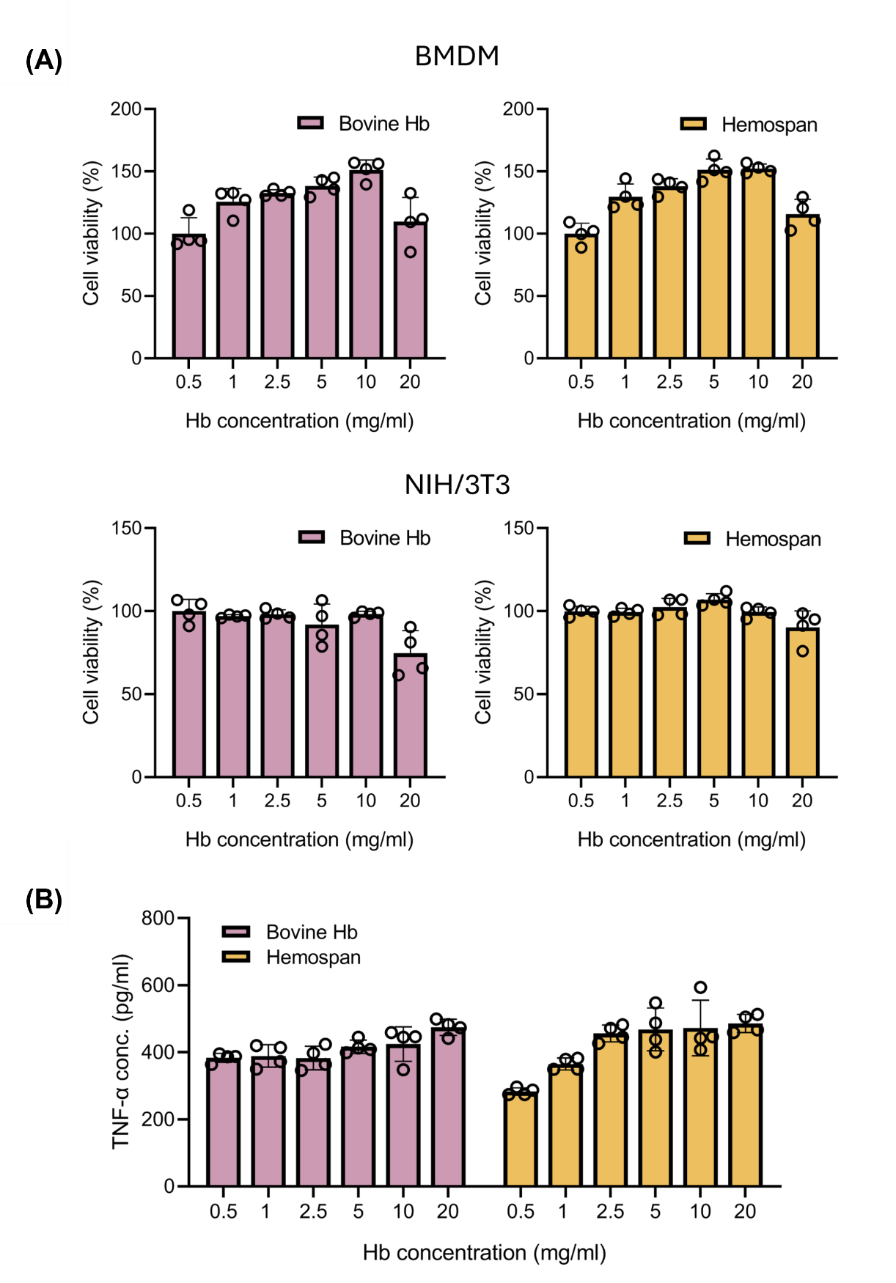


**Supplementary Figure 1. In vitro cytotoxicity assessment of bovine Hb and Hemospan.** (A) Cell viability evaluation of bovine Hb and Hemospan in BMDMs and NIH/3T3 cells after 24 hr incubation, assessed using a CCK-8 assay. (B) Measurement of pro-inflammatory TNF-α secretion from BMDMs treated with bovine Hb and Hemospan by ELISA. Bovine Hb and Hemospan were applied at Hb-equivalent concentrations matching those of Hb@Dex-NGs (0.5, 1, 2.5, 5, 10, and 20 mg/mL). For Hemospan, the corresponding polyethylene glycol (PEG) concentrations were 0.8, 1.6, 4, 8, 16, and 32 mg/mL, respectively.
